# An overview of the potential anticancer properties of cardamonin

**DOI:** 10.37349/etat.2020.00026

**Published:** 2020-12-28

**Authors:** Shanaya Ramchandani, Irum Naz, Namrata Dhudha, Manoj Garg

**Affiliations:** 1 Department of Pharmacology Biomedicine, the University of Melbourne, Parkville Victoria 3010, Australia; 2 Department of Biochemistry, Quaid-i-Azam University, Higher Education Commission of Pakistan, Islamabad 44000, Pakistan; 3 Department of Biotechnology and Microbiology, School of Sciences, Noida International University, Noida 201301, India; 4 Amity Institute of Molecular Medicine and Stem cell Research (AIMMSCR), Amity University Uttar Pradesh, Noida 201313, India; National University of Singapore, Singapore

**Keywords:** Cancer, cardamonin, chalcone, apoptosis, proliferation, Wnt/β-catenin, NF-κB, PI3K/Akt

## Abstract

Cancer is one of the leading causes of mortality, contributing to 9.6 million deaths globally in 2018 alone. Although several cancer treatments exist, they are often associated with severe side effects and high toxicities, leaving room for significant advancements to be made in the field. In recent years, several phytochemicals from plants and natural bioresources have been extracted and tested against various human malignancies using both *in vitro* and *in vivo* preclinical model systems. Cardamonin, a chalcone extracted from the *Alpinia* species, is an example of a natural therapeutic agent that has anti-cancer and anti-inflammatory effects against human cancer cell lines, including breast, lung, colon, and gastric, in both *in vitro* culture systems as well as xenograft mouse models. Earlier, cardamonin was used as a natural medicine against stomach related issues, diarrhea, insulin resistance, nephroprotection against cisplatin treatment, vasorelaxant and antinociceptive. The compound is well-known to inhibit proliferation, migration, invasion, and induce apoptosis, through the involvement of Wnt/β-catenin, NF-κB, and PI3K/Akt pathways. The good biosafety and pharmacokinetic profiling of cardamonin satisfy it as an attractive molecule for the development of an anticancer agent. The present review has summarized the chemo-preventive ability of cardamonin as an anticancer agent against numerous human malignancies.

## Introduction

Cancer is the second-leading cause of death worldwide, following cardiovascular disease, and accounts for 1 in every 6 deaths [1–6]. According to the National Cancer Institute, there is nearly a 40% chance of men and women developing cancer at some point during their lifetime [1, 2]. The most common cancers include breast, lung, liver, ovarian, colorectal, and prostate [1–6]. Several chemotherapeutic treatments exist for these malignancies. However, these treatments are not effective in generating long-term remission and improved quality of life [1–6]. The majority of cancer deaths occur in lower-income families, due to the high cost of treatment options in clinics [1–6]. Therefore, substantial effort has been explored to find out alternative, cost-effective, less toxic treatments that are widely available. The products comprising these options can be derived from natural resources and their compounds [7–11].

Cancer is a disease characterized by abnormal and uncontrolled cellular proliferation, forming malignant tumors that can spread or metastasize to other parts of the body [8, 12]. Cancer cells can grow rapidly, invade, and migrate through the lymphatic and circulatory systems [8, 12]. Hanahan and Weinberg have proposed several hallmarks of cancer such as proliferative advantage, the ability to invade, the ability to migrate from the primary site, metastasis, selective growth, altered stress responses, and immune modulation [12]. Several risk factors promote these hallmarks, including smoking, obesity, alcohol, aging, genetic dispositions, exposure to UV radiation, chemicals, and some viral infections, such as human papillomavirus and hepatitis B virus. These risk factors have either individual roles or can work in conjunction with one another to promote the growth and proliferation of cancer cells [7, 12]. Numerous cancer treatments, such as radiation and chemotherapy, have been put into place. However, these are often associated with detrimental side effects, such as erosion of the digestive lining, loss of test buds, liver toxicity, renal injury as well as hair loss. Most of the existing anti-cancer drugs have lower specificity and selectivity towards cancer cells. Hence, they affect normal living cells. Additionally, many of these drugs are also toxic, which leads to high mortality rates [13–14].

As a result, several natural compounds have been tested against cancer cell lines in preclinical and clinical phases, including herbs, spices, pulses, and nuts. In fact, several successful anticancer drugs such as docetaxel, paclitaxel, topotecan have been isolated from plants and herbs. These bioresources are rich sources of phytochemical agents that have been displayed to possess therapeutic properties against human cancer cell lines. For example, natural drugs such as casticin, extracted from the *Fructus viticus* species have been used as an analgesic for menstrual pain in females. Also, casticin was reported with antineoplastic effects in cancer cell lines and mouse models [15]. Zerumbone, derived from *Z. zerumbut*, is known for its biochemical properties as well as antiproliferative activity against human malignancies drawing attention for further research by the scientific community [16].

Likewise, cardamonin, a naturally occurring chalcone isolated from several cardamom plants (specifically, *Alpinia katsumadai* and *Alpinia conchigera*), have been used as a treatment for numerous ailments in South America [17–18]. It is a component of the *Myrtacae* family that has been conventionally used to treat stomach issues and diarrhea. In recent years, significant emphasis has been placed on the role of cardamonin as an anticancer agent. Cardamonin mediates inflammation, targets pathogens, and modulates the immune system [19–20]. Numerous studies have been conducted on cardamonin role against lung, breast, prostate, and colon cancer, *in vitro* and *in vivo*. Interestingly, cardamonin has been shown to be effective against aggressive deadlier cancers including glioblastoma, multiple myeloma, melanoma, nasopharyngeal carcinomas (NPCs), and ovarian [17–28]. Also, reports have shown promising effects of the compound, particularly through the Wnt and nuclear factor kappa-light-chain enhancer (NF-κB) pathways. This review details the potential anti-cancer activity of cardamonin along with molecular mechanisms of action in human cancers.

## Chemistry

**Figure 1.**
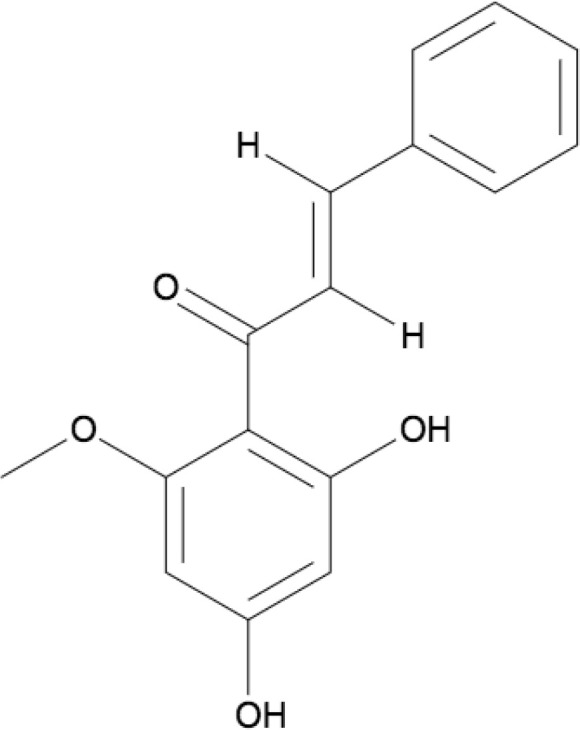
Molecular structure of cardamonin (ChemDraw)

Cardamonin (CARD/CD) is chemically known as (E)-2′,4′-dihydroxy-6′-methoxy-chalcone which is derived from a group of aromatic enones which belong to a family of flavonoids, contributing to the yellow-color of pigmented plants. Many plants, such as *Alpinia katsumadai* and *Alpinia conchigera*, contain flavonoids that are responsible for anti-proliferative and apoptotic mechanisms. Cardamonin is an isolated chalcone and the molecular formula is C_16_H_14_O_4_ (Figure 1). Analogues of cardamonin include 4,4’-dihydroxylchalcone (DHC) and 4,4’-dihydroxy-2’-methoxychalcone (DHMC), which were used as substituents in some of the studies [29]. Naturally, chalcones are found in the leaf, fruit, or root of numerous plant species. Also, several chalcones can be artificially synthesized in laboratories through the acid-base catalyzation of an aldehyde or ketone for preclinical studies [30]. The molecular structure of cardamonin is depicted in Figure 1.

## Anticancer effects of cardamonin *in vitro* cancer cell lines

**Figure 2.**
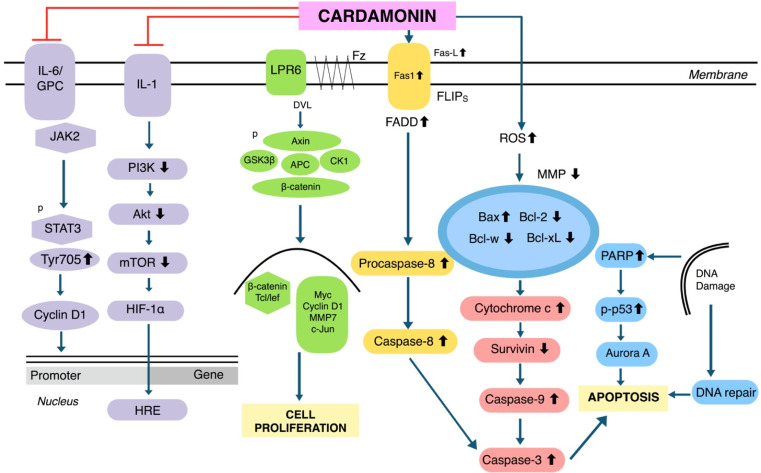
Molecular pathways of cardamonin

Cardamonin was reported to be effective in several human cancers including breast, cervical, colon, gastric, lung, ovarian, prostate, glioblastoma, leukemia, melanoma, and multiple myeloma. A summary of the effects of cardamonin on each of these malignancies is provided below, along with the half-maximal inhibitory concentrations (IC_50_ values) for respective cell lines (Table 1). The primary mechanism for cardamonin is the mammalian target of rapamycin (mTOR) signaling pathway, which plays a crucial role in autophagy (the removal of damaged cells), inhibiting proliferation and regulating cell metabolism (Figure 2). Cardamonin can upregulate pro-apoptotic proteins such as Bcl-2 associated X protein (Bax) and caspase-3, and downregulate anti-apoptotic molecules such as B-cell lymphoma 2 (Bcl-2), thus inducing apoptosis in various cell lines (Figure 2).

**Table 1.** Anticancer effects of cardamonin *in vitro*

**Cancers**	**Cell line**	**Phenotypic effects**	**Mechanisms of action**	**References**
**Breast cancer**	MDA-MB-231, MCF-7	Induced apoptosis and G2/M cell cycle arrest; inhibited proliferation	↑ROS, ↑FOXO3a, ↑p21, ↑p27, ↑Bim, ↓Cyclin D1, ↑caspase-3	[17]
	BT-549	Induced apoptosis and cell cycle arrest; decreased invasion and migration	↑E-cadherin, ↓Snail, ↓Slug, ↓Vimentin, ↑GSK3B, ↓EMT, ↓β-catenin	[20]
	MDA-MB-231	ROS-induced apoptosis	↓HIF-1a, ↓mTOR/p70SK, ↑OXPHOS, ↓Nrf2, ↑ROS	[36]
	Drug-resistant CSC’s	Suppresses existing cells and prevents the formation of new cells	↑IL-6, ↑IL-8, ↑MCP-1	[37]
**Cervical cancer**	HeLa	Inhibited cell proliferation	↓mTOR, ↓S6K1, ↓raptor	[41]
**Colon cancer**	HCT-116	Suppressed growth; induced apoptosis	↑caspase-3, ↑caspase-9, ↑Bax, ↓c-Myc, ↓4k cyclin E, ↓p50, ↓NF-κB p65, ↓Bcl-2	[42, 43]
**Gastric cancer**	AGS	Inhibited cell proliferation and migration; induced apoptosis, cell cycle arrest at G0/M phase	↑E-cadherin, ↓Snail, ↓Slug, ↓Vimentin, ↓Bcl-2, ↑Bax, ↓caspase-3, ↓CDK1, ↓cyclin B1, ↑p21	[49]
	BCG-823 and BCG-823/5-FU	Enhanced chemosensitivity of 5-FU; induced apoptosis, and cell cycle arrest	↓p-glycoprotein, ↓β-catenin, ↓TCF-4, ↓Wnt/β-catenin	[50]
**Glioblastoma**	CD133 + GSCs	Inhibited proliferation; induced apoptosis	↓STAT3, ↓Bcl-2, ↓Bcl-L, ↓Mcl-1, ↓Survivin, ↓VEGF	[52]
**Leukemia**	WEHI-3	Decreased cell viability; induced apoptosis	↑ROS, ↑Ca^2+^, ↓ΔΨm, ↑caspase-3, ↑caspase-8, ↑caspase-9, ↓Bcl-2, ↑Bax, ↑cytochrome c, ↑AIF, ↑Endo G, ↑GRP78, ↑caspase-12, ↑Fas, ↑Fas-ligand, ↑FADD, ↑DAP, ↑TMBIB4, ↑ATG5, ↓DDIT3, ↓DDIT4, ↓BAG6, ↓BCL2L13, ↓BRAT1	[53]
**Lung cancer**	A549 and H460	Induced apoptosis, G2/M cell cycle arrest; reduced cell migration, and invasion	↑caspase-3, ↑Bax, ↓Bcl-2, ↓cyclin D1, ↓CDK4, ↓PI3K, ↓Akt, ↓mTOR	[60]
	LLC	Reduced proliferation, invasion, and migration	↓Snail, ↑E-cadherin, ↓mTOR, ↓S6K1, ↓NF-κB	[62]
	A549 and NCI-H460	Suppressed NF-κB activation	↓NF-κB	[63]
	A549	Inhibited proliferation; induced cell cycle arrest, and apoptosis	↓mTOR, ↓p70S6K	[64]
**Melanoma**	A375	Induced apoptosis and increased cytotoxicity	↑caspase-3, ↑PARP	[65]
	M14 and A375	Inhibited cell viability and migration; reduced cell density; induced apoptosis	↓Bcl-2, ↑Bax, ↑cleaved caspase-8, ↑cleaved caspase-9, ↑PARP, ↓NF-κB p65	[61]
**Multiple myeloma**	Myeloma cells	Suppressed cell viability; induced apoptosis	↑PARP, ↓Bcl-2, ↑Bax, ↑caspase-3, ↓NF-κB, ↓IKK, ↓IkBa, ↓ICAM-1, ↓COX-2, ↓VEGF	[70, 71]
	Myeloma cells	Induced apoptosis and cell cycle arrest; controlled proliferation	-	[70, 71]
**Ovarian cancer**	SKOV3 and A2780	Inhibited proliferation; induced apoptosis	↓Bcl-2, ↓XIAP, ↓survivin, ↓mTOR	[72]
	SKOV3	Inhibited proliferation; enhanced autophagy	↓Lactate, ↓ATP, ↓HK, ↓LDH, ↑LC3-II, ↓mTORC1, ↓H2K, ↑AMPK	[73]
**Prostate cancer**	PC-3	Decreased cell proliferation, growth, and viability; induced apoptosis	↓STAT3, ↓NF-κB1	[74, 75]

FOXO3a: Forkhead box O3; ROS: reactive oxygen species; HIF-1a: hypoxia-inducible factor-1a; Nrf2: NF-E2 related factor 2; OXPHOS: mitochondrial oxidative phosphorylation; CSC: cancer stem cell; STAT3: signal transducer and activator of transcription 3; IL-6: interleukin 6; IL-8: interleukin 8; EMT: epithelial-mesenchymal transition; GSC: glioblastoma stem cells; ATG5: autophagy-related 5; DDIT3: DNA-damage inducible transcript 3; LCC: Lewis lung carcinoma; S6K1: S56 kinase 1; PARP: poly(ADP-ribose) polymerase; LC3-II: light chain 3-II; Akt: protein kinase B; VEGF: vascular endothelial growth factor; XIAP: X-linked inhibitor of apoptosis protein

### Breast carcinoma

Breast cancer is the second-leading cause of cancer-related mortalities in women worldwide [31–34]. In 2019, approximately 42, 000 breast cancer mortalities occurred in the United States alone [35]. In a study by Kong et al. [17], the pretreatment of MDA-MB231 and MCF-7 cells with cardamonin resulted in the induction of G2/M cell cycle arrest, through the downregulation of cyclin D1, and apoptosis. Through the upregulation of c-Jun FOS-binding proteins-a family of protein kinases responsible for an apoptosis-FOXO3a translocation was enhanced, along with its downstream regulatory proteins: p21, p27, and Bim. Additionally, cardamonin increases the expression of activated caspase-3 to induce cell death [17]. Furthermore, the Wnt/β-catenin pathway was downregulated through the inhibition of phosphorylated glycogen synthase kinases (GSKs) through Akt [20]. Cardamonin has been shown to suppress the growth of breast cancer cell lines *in vitro*. A study conducted by Jin et al. [36], on MDA-MB-231 breast cancer cells highlighted the ability of cardamonin to mediate ROS-induced apoptosis by decreasing the expression of critical cell metabolic modulators, such as HIF-1a. The IC_50_ values of MDA-MB-231 are 52.885 μM, 33.981 μM, and 33.981 μM for 24 h, 48 h, and 72 h, respectively [36]. The natural flavone inhibited mRNA levels by suppressing mTOR/p70S6K levels. Also, mTOR regulates the invasion and motility of cancer cells. Through the retardation of these targets, cardamonin can suppress cell proliferation in various malignancies [36]. Moreover, Nrf2-dependent proteins were downregulated, which further increased ROS levels. Pretreatment with cardamonin inhibited glycolysis and increased OXPHOS signaling [36]. In another study, cardamonin suppressed the growth of drug-resistant breast CSCs and prevented the self-renewal of CSCs through the inhibition of NF-κB and STAT3 pathways. In CSCs, cardamonin has displayed promising effects when used in combination with chemotherapeutic agents through the downregulation of IL-6 and IL-8 pro-inflammatory factors [37]. Cardamonin has been reported to reduce the invasion and migration of triple-negative breast cancer cells (TNBC, BT-549 cells) via reversal of EMT by increasing the levels of E-cadherin [37–38]. Cardamonin also caused the downregulation of Snail, Slug, and Vimentin, crucial mesenchymal markers [37–39]. Through these mechanisms, cardamonin has been proven to have inhibitory effects against breast cancer cell lines.

### Cervical carcinoma

Cervical cancer is deadly cancer occurring in women and has a considerably high mortality rate especially in low- and middle-class income groups, due to the lack of awareness and affordable treatment [40]. It is, therefore, imperative, that natural and inexpensive treatments are formulated for cervical cancer patients. Additionally, several cancers, such as cervical and breast cancers, are resistant to mTOR inhibitors. In a study, HeLa cervical cancer cells, which were resistant to high doses of mTOR-inhibitors have been treated with cardamonin. Cardamonin treatment was efficient enough to attenuate cell proliferation through reduced phosphorylation of mTOR and S6K1 in mTOR inhibitor-resistant HeLa cells [41].

### Colon carcinoma

Colon cancer is the third most prevalent cancer in the United States in both men and women. The death rate of colon cancer has been noticed to be reduced over the last decade; however, it is still expected to cause around 55, 000 deaths in 2020. The combination of cardamonin with other chemotherapeutic agents such as 5-FU was reported to reduce the growth of chemo-resistant HCT-116 colon cancer cells whiling inducing apoptosis [42]. The compound has been shown to activate caspase-3 and caspase-9, increased expression of pro-apoptotic Bax, decreased expression of anti-apoptotic Bcl-2, and regulated c-Myc, octamer-binding transcription factor 4k (OCT4), Cyclin E, including cell cycle arrest [42]. Moreover, cardamonin downregulated testes-specific protease 50 and NF-κB p65 expression [42]. In another study, cardamonin inhibited β-catenin response transcription in SW480 colon cancer cells and induced G2/M cell cycle arrest. The compound decreased levels of β-catenin, on which catenin response transcription (CRT) is highly dependent, and inhibited cyclin D1 and c-Myc [43]. Furthermore, it inhibited β-catenin/TCF-4, a downstream protein of β-catenin. Through these mechanisms of action, cardamonin expressed its therapeutic potential on cells carrying mutations in the *APC* genes, without posing any threat on the levels of mRNA [43].

### Gastric cancer

Being the fifth most commonly diagnosed cancer, and the third deadliest cancer globally, gastric cancer has a survival rate of less than 5% [44–48]. Cardamonin has proven to be an effective treatment in AGS cells by inhibiting cell proliferation, migration, and induced apoptosis. Cardamonin downregulated the expression of anti-apoptotic Bcl-2 protein while upregulating Bax and caspase-3 levels. Furthermore, cardamonin was reported to increase of p21, and decrease CDK1, cyclin B1, and CDC25c to block the AGS cells in the G0/G1 phase of the cell cycle [49]. Cardamonin also suppressed the expression of the EMT markers including Snail, Slug, and Vimentin, and increased E-cadherin expression levels. Another study emphasized the useful effect of cardamonin when used in combination with 5-FU. In this study, cardamonin was displayed to enhance the chemosensitivity of BCG-823 and BCG-823/5-FU cells through the inhibition of the Wnt/β-catenin pathway [50]. This led to the mediation of TCF4 transcription and resulted in apoptosis and cell cycle arrest *in vitro* [50]. These two studies indicate that cardamonin exerts the anti-cancer effect either alone or in conjunction with other drugs.

### Glioblastoma

Glioblastoma is a type of brain malignancy, which commonly occurs in adults and is extremely aggressive due to its ability to metastasize and resistance to chemotherapy [51]. Cardamonin was shown to sensitize the CD133+ GSCs *in vitro*. Cardamonin inhibited the expression of Bcl-2, Bcl-xL, and Mcl-1 proteins, and increased Bax levels, resulting in decreased proliferation and robust apoptosis. Levels of survivin and VEGF were also decreased through docking in nuclear STAT3 activation. When docked, these proteins have high binding energies of −10.78 kcal/moL, resulting in a highly energized exothermic process [52]. Therefore, the inhibition of STAT3 activation via cardamonin led to a decrease in the proliferative properties of CD133+ cells in glioblastoma.

### Leukemia

Cardamonin has shown promising anti-leukemic activity against leukemia cells. A study conducted by Liao et al. [53], investigated the effect of cardamonin on WEHI-3 mice cells *in vitro* and found that the compound decreased cell viability and enhanced apoptosis through the mediation of numerous proteins. Firstly, ROS activity and the production of Ca^2+^ was increased, resulting in ROS-induced apoptosis. Mitochondrial membrane potential (MMP) was downregulated, while caspase-3, caspase-8, and caspase-9 were upregulated [53]. An increase in pro-apoptotic factors including cytochrome C, AIF, Bax, Endo G release, and caspase-12 was observed, as well as a decrease in anti-apoptotic Bcl-2 with pretreatment with cardamonin [53]. Apoptotic cell death was induced through an increase in Fas, FaDD, and Fas-ligand expression. Furthermore, cardamonin treatment was associated with increased expression of Bax inhibitor motif-containing 4, ATG5, DDIT3, DDIT4, and BRCA1-associated ATM activator 1. This resulted in a change in the morphology of WEHI-3 cells, in turn, causing programmed cell death [53].

### Lung cancer

Globally, lung cancer accounts for one-fourth of cancer-related deaths [54–59]. Cardamonin has provided promising anticancer efficacy against lung cancer *in vitro*. In non-small-cell lung cancer (NSCLC) A549 and H460 cells, cardamonin activated caspase-3, increased Bax levels, decreased Bcl-2 levels, thus inducing apoptosis [60]. Furthermore, the downregulation of cyclin D1/CDK4 levels resulted in the induction of cell cycle arrest in the G2/M phase. Moreover, treatment with cardamonin also reversed EMT transition and regulated several downstream factors including PI3K and mTOR signaling [60]. Through these mechanisms, the compound suppressed the ability of these NSCLC to invade and migrate. Several previous studies have established that cardamonin is highly associated with mTOR inhibition [60]. Similarly, in a second study, the chalcone generated a reduction in proliferative, invasionary, and migratory properties of LLC cells through the attenuation of Snail and other downstream targets including ribosomal S6K1 and the inhibition of NF-κB [61]. Additionally, levels of E-cadherin were upregulated and intracellular adhesion and separation of cancerous cells from tissues, i.e. the ability to metastasize, were decreased through the regulation of mTOR pathways [62]. Another study has utilized two analogs of cardamonin, DHC, and DHMC against A549 and NCI-H460 lung cancer cells [62]. The activity of these analogs downregulated the activation of NF-κB and induced apoptosis [62]. In a final study, cardamonin was reported to attenuate cell proliferation and induce cell cycle arrest in A549 lung cancer cell lines. Cardamonin inhibited p70S6K and mTOR activation but did not affect the levels of FKBP12 and IL-2 [63]. Nevertheless, the expression of these proteins was decreased through binding with rapamycin [64]. Through these four studies, there is strong evidence that cardamonin can suppress the growth of lung cancer malignancies with experiments performed on *in vitro* cell lines.

### Melanoma

Melanoma is the deadliest type of skin cancer and accounts for over three-fourths of skin cancer mortalities [61, 65]. Several natural remedies, which involve using plant constituents, are being tested against melanomas. Melanomas can metastasize easily through lymph nodes and treatment options are relatively ineffective. Consequently, dietary chalcone was used against A375 malignant cell lines and IC_50_ values were 3.98 μM for 48 h and 2.43 μM for 96 h, respectively. Further, no significant toxicity of cardamonin against normal human epidermal melanocytes was reported [65]. Cardamonin can induce apoptosis of A375 cells through an increase in caspase-3 and PARP cleavage activity. These data displayed that cardamonin has a stronger cytotoxic effect on melanoma cells compared to healthy cells *in vitro* [65]. Another study showed that cardamonin reduced the cell viability in a concentration-dependent manner in melanoma M14 and A375 cell lines [61] by enhancing cell death through the upregulation of Bax, cleaved caspase-8, -9, and PARP, and downregulation of Bcl-2 factors. Furthermore, cardamonin can inhibit the NF-κB pathway intrinsically and extrinsically as levels of p65 were decreased [61].

### Multiple myeloma

Multiple myeloma is another type of blood cancer occurring in plasma cells [66–71]. The therapeutic potential of cardamonin was tested against multiple myeloma cell lines. Pretreatment with cardamonin increased caspase-3 and PARP activation, and caused the suppression of numerous anti-apoptotic proteins, thus, inducing apoptosis and reducing cell viability in human myeloma cells [70–71]. Cardamonin also reduced the activation of the NF-κB pathway, through an increase in IKK and IkBA phosphorylation. Also, the levels of ICAM-1, COX-2, and VEFG were attenuated [70–71]. Moreover, cardamonin was found to suppress cell proliferation and induced apoptosis in human myeloma cells and exhibited several anti-myeloma effects, including reduced cell viability and blocking of specific proteins involved in the cell cycle, leading to eventual arrest [70–71].

### Ovarian cancer

Ovarian cancer is the most common type of gynecological malignancy worldwide. The 5-year survival rate of ovarian cancer is between 20–47%. Platinum-based chemotherapies are the best treatment against ovarian cancer in the clinics. Cardamonin has been associated with anti-proliferative effects against SKOV3 and A2780 ovarian cancer cells by inducing apoptosis, attenuating the levels of Bcl-2, survivin, and mTOR cascade. Additionally, cardamonin induced cell cycle arrest in the G2/M phase. Also, their study proved that the combined treatment of cardamonin along with cisplatin has a stronger and synergistic anti-cancer effect [72]. Another study, conducted on SKOV3 ovarian cancer cells, demonstrated the antiproliferative properties of cardamonin. The compound also enhanced autophagy or the removal of damaged cells by suppressing mTORC1 pathways and H2K expression. Cardamonin was reported to decrease the production of ATP and lactate along with upregulated microtubule-associated protein 1 LC3-II, and lysosome-associated membrane protein 1 upregulation [73].

### Prostate cancer

The occurrence of prostate cancer is extremely high, affecting one in every nine men worldwide, with older men being more at risk [74–75]. Zhang et al. [74], have reported that cardamonin can efficiently block the STAT3 signaling cascade to repress the growth and viability of prostate cancer cells. Pascoal et al. [75], have demonstrated the effect of the isolated chalcone on PC-3 cell lines. This study showed that the cardamonin decreased the proliferation and viability of PC-3 cells via decreased expression of NF-κB.

## Anti-tumor efficacy of the cardamonin *in vivo* using the murine model system

Aside from *in vitro* studies, the effects of cardamonin have been further investigated in preclinical studies, particularly in murine models (Table 2). A study conducted by Jin et al. [36], has displayed that co-administration of cardamonin and 5-FU against MDA-MB-231 xenograft models caused decreased tumor growth with an increased cleaved caspase-3 and Bax/Bcl-2 ratio. This study reported that the combination had minimal effect on the overall body weight of the mice. Cardamonin treatment has been found to attenuate the levels of HIF-1α and LDHA and resulted in decreased tumor angiogenesis [36]. Interestingly, in another study in breast cancer, combined treatment of cardamonin along with other chemotherapeutic drugs was found to reduce the growth of CSCs and tumor growth. Cardamonin can reduce xenograft tumor growth in TNBCs at a dose of 5 mg/kg [37]. The 5-FU along with chalcone was noticed with the marked reduction of neoplasm growth in chemo-resistant gastric cancer BCG-823/5-FU cells in BALB/c xenograft nude mice, confirming *in vitro* findings [51]. An extensive study was carried out *in vivo* to test the ability of cardamonin against leukemia affected BALB/c mice. The mice were divided into four groups, including negative and positive controls; the groups were treated with 1 mg/kg and 5 mg/kg doses of the compound for two weeks. The results indicated that CD3, CD11b, and Mac-3 were downregulated, however, CD19 proteins were upregulated [53]. Cardamonin also enhanced immune system capacity by increasing the phagocytic ability of macrophages in peripheral blood mononuclear cells, serving as a therapeutic agent for leukemia patients preclinically [53]. Furthermore, cardamonin reduced tumoral growth and induced apoptosis in NSCLC A549 and H460 lung cancer cells, by decreasing expression rates of Ki-67 and phosphorylated Akt and mTOR. Tumor growth and metastatic abilities were reduced by the PI3K/Akt/mTOR pathway [60, 62]. Results from another *in vivo* lung cancer study conducted on LLC cells indicated that both lung cancer metastasis and tumoral growth were suppressed through the inhibition of mTOR, as rapamycin prevented movement to distant nodes [60, 62].

**Table 2.** Anticancer effects of cardamonin *in vivo*

**Cancers**	**Cell line**	**Phenotypic effects**	**Mechanisms of action**	**References**
**Breast cancer**	MDA-MB-231	Inhibitory effects on tumor angiogenesis	↓Bcl-2, ↑Bax, ↑caspase-3, ↓HIF-1a, ↓LDHA	[36]
	Drug-resistant CSC’s	Inhibited tumor growth and volume	-	[37]
**Gastric cancer**	BCG-823/5-FU	Reduced tumor growth	-	[50]
**Leukemia**	BALB/c mice	-	↓CD3, ↓CD11b, ↓Mac-3, ↑CD19	[53]
**Lung cancer**	NSCLC A549, H460	Reduced cell proliferation and metastatic abilities	↓Ki-67, ↓mTOR, ↓Akt	[60]
	LLC cells	Suppressed lung metastasis and tumoral growth	↓mTOR	[62]

## Biosafety profiling of cardamonin

Berning and colleagues have shown that cardamonin represses the growth of melanoma cells without affecting the normal cells [65]. Few studies describe the biosafety profile of cardamonin. Their studies showed that few data are available on its absorption, distribution, metabolism as well as excretion of the cardamonin [76–77]. Jaiswal et al. [78–79], have reported that cardamonin is less soluble as well as a high rate of permeability/penetration. Cardamonin has a moderate ability to binds with plasma proteins as well as its lower uptake in red blood cells. Cardamonin was displayed to be excreted from mice in the feces in the major amount whereas a small amount was present in urine. Cardamonin displayed anti-tumor potential but its limited bioavailability suppresses the effectiveness of cardamonin [78–79]. The bioavailability of cardamonin was reported to gender-biased in the experiments performed on rats. Therefore, more studies are required to improve the bioavailability in near future.

## Conclusions and future perspective

Cancer belongs to one of the greatest causes of mortality worldwide, responsible for 9.6 million deaths in 2018. Although there are numerous advancements in cancer research, methods of cancer diagnosis, development, and approval of the new drugs for treatment, still the scope for improvement to develop better drugs with less toxic and side effects are required. One of the approaches can be the screening of natural compounds for their anti-tumor activity as several clinical grades and FDA approved drugs were extracted from natural bioresources. Cardamonin has shown anti-tumor activity in preclinical studies by targeting numerous signaling pathways such as Wnt/β-catenin, NF-κB, and STAT3. Attenuation of these signaling pathways by cardamonin induces cell cycle arrest and cell death either alone or in combination with other chemotherapeutic agents. Interestingly, cardamonin enhances the antitumor activity in drug-resistant cells in several malignancies. Moreover, cardamonin showed promising anti-cancer efficacy in xenograft murine models against breast, gastric, lung, and leukemia. These data suggest that cardamonin may be used in combination with existing drugs in several human malignancies to provide a strong foundation for clinical trials in the near future, especially in murine models. Also, the focus of future research on cardamonin will be to check its anti-inflammatory activity and association with immune response. We are hopeful that future studies will provide a strong basis for its clinical trials to determining whether cardamonin possesses the same antineoplastic effects in humans and the doses at which the compound is effective.

## Abbreviations

Akt: protein kinase B
ATG5: autophagy-related 5
Bax: Bcl-2 associated X protein
Bcl-2: B-cell lymphoma 2
CSC: cancer stem cell
DDIT3: DNA-damage inducible transcript 3
DHC: 4,4’-dihydroxylchalcone
DHMC: 4,4’-dihydroxy-2’-methoxychalcone
EMT: epithelial-mesenchymal transition
FOXO3a: Forkhead box O3
GSC: glioblastoma stem cells
GSK: glycogen synthase kinases
HIF-1a: hypoxia-inducible factor-1a
IC_50_: half-maximal inhibitory concentration
IL-6: interleukin 6
IL-8: interleukin 8
LC3-II: light chain 3-II
LLC: Lewis lung carcinoma
mTOR: mammalian target of rapamycin
NF-κB: nuclear factor kappa-light-chain enhancer
Nrf-2: NF-E2 related factor 2
NSCLC: non-small-cell lung cancer
OXPHOS: mitochondrial oxidative phosphorylation
PARP: poly(ADP-ribose) polymerase
ROS: reactive oxygen species
S6K1: S56 kinase 1
STAT3: signal transducer and activator of transcription 3
TNBC: triple-negative breast cancer
VEGF: vascular endothelial growth factor

## Declarations

### Copyright

© The Author(s) 2020.
